# Editorial: The Brain Under Fatigue

**DOI:** 10.3389/fnins.2022.945527

**Published:** 2022-06-03

**Authors:** Haijing Niu, Dong Ming, Christos Papadelis, George Alexandrakis

**Affiliations:** ^1^State Key Laboratory of Cognitive Neuroscience and Learning, Beijing Normal University, Beijing, China; ^2^Department of Biomedical Engineering, Tianjin University, Tianjin, China; ^3^Cook Children's Medical Center, Fort Worth, TX, United States; ^4^Department of Pediatrics, School of Medicine, Texas Christian University, Fort Worth, TX, United States; ^5^Bioengineering Department, University of Texas at Arlington, Arlington, TX, United States

**Keywords:** central fatigue, peripheral fatigue, brain imaging, EEG, isometric contraction, virtual reality, myalgic encephalomyelitis/chronic fatigue syndrome (ME/CFS), cardiorespiratory diseases

Does what is colloquially known as “mind over matter” have a neurological basis? If our body's muscles start feeling tired (peripheral fatigue), at what point does the brain start getting tired too (central fatigue), while we try to power through a task? As it turns out these innocuous-looking questions are challenging to study in the lab. The purpose of this Research Topic was to collect articles that capture an, albeit incomplete, snapshot of the state of the art in this emerging field that studies what it means to feel fatigued in health and disease.

This Research Topic includes six articles in total. The first two articles studied the effects of different physical activities on the temporal evolution of peripheral and central fatigue in healthy adults, across the lifespan. The first study (Suviseshamuthu et al.) explored how EEG spectra were modulated by an intermittent motor task, often involved in activities of daily living. Specifically, 14 participants (median age 51.5 years; age range 26–72 years; 6 males) repeated elbow flexion at 40% maximum voluntary contraction by following visual cues until subjective exhaustion. The power spectral density bands related to sensorimotor processing scaled with increasing physical effort, but no age and gender-based differences were found. Another study (Wylie et al.) examined 43 healthy individuals across age (20–55+) and gender for both perceived fatigue (self-reported) overall and while performing a fatiguing task inside an fMRI scanner. There was no correlation between how fatigued individuals felt overall and their fMRI patterns. Interestingly, older persons (55+) reported less fatigue during the task. Neuroimaging data highlighted the role of middle frontal areas across age: younger individuals were able to recruit these areas to combat fatigue, but older individuals did not recruit them while powering through, which may have contributed to lower fatigue perception.

Two other articles in this Research Topic were focused on how younger individuals fatigue while performing physical or virtual-reality (VR) tasks that mimic athletic or gaming activities. This first study (Wang et al.) examined the effects of transcranial direct current stimulation (tDCS) on the time to exhaustion in relation to muscle activities and corticomuscular coupling of agonist and antagonist muscles in the elbow during a sustained isometric fatiguing contraction. It was found that tDCS significantly increased time to exhaustion relative to control male subjects (10 per group) receiving sham stimulation. Task improvement was related to increased neuromuscular efficiency as reflected by decrease of antagonistic muscle coactivation activities (EMG), correlating with increased relative cortical activation in sensorimotor regions post-stimulation (EEG). The second study (Nürnberger et al.) examined how visually induced motion sickness was created by VR devices. Their study used EEG in a VR environment for 14 healthy subjects (8 male) who were exposed to increasing levels of mismatch between vestibular and visual information. They found that with increasing motion sickness, the proportion of slow EEG waves (1–10 Hz) increased, especially in temporo-occipital regions, and that there was a decrease in information flow between most brain areas, a form of mental fatigue. The researchers hypothesized that a progressive reduction of information flow in the brain with increasing vestibular-visual mismatch originated from the brain's efforts to transform an unstable model into a stable one with minimal contradictory information.

Lastly, two papers in this Research Topic examined how different medical conditions could reduce exercise capacity, and by extension the ability to perform activities of daily living, by exacerbating fatigue. In the first of these papers (Rayhan and Baraniuk) subjects with myalgic encephalomyelitis/chronic fatigue syndrome (ME/CFS) and control subjects (34 ME/CFS and 24 controls, females 24 and 10, respectively) performed two submaximal bicycle exercise stress tests on consecutive days, bracketed by fMRI and other assessments, to identify objective changes of exercise-induced symptom exacerbation and cognitive dysfunction. The main finding of this study was that the dynamic increase in activation of the anterior default mode network node after exercise may be a biomarker of post-exertional malaise and symptom exacerbation in CFS. Another article (Marillier et al.) reviewed studies performed to date based on the rationale that the brain is the organ limiting tolerance to physical exertion in patients with cardiorespiratory diseases. The authors compiled comprehensively the mechanistic insights from the studies published to date for establishing the contribution of central and/or supraspinal mechanisms in limiting exercise tolerance in major cardiorespiratory diseases. They concluded that improvements in exercise tolerance after exercise reconditioning are typically lost over time in patients with chronic cardiorespiratory diseases and that future research should also focus on the best strategies to promote sustainable behavioral changes.

A birds-eye view of this article collection suggests that the field of fatigue is very broad indeed, which makes drawing generalizable conclusions challenging and leads us to a few key questions and suggestions for future directions:

(1) Though we have all felt very tired, or realized we are approaching exhaustion, how do these verbal expressions translate into scientific metrics of fatigue? Can we define quantitative biomarkers of central fatigue and distinguish them from peripheral fatigue with age and gender? It is challenging to connect data across different studies using today's subjective scales. The rapidly expanding use of wearables provides opportunities for non-subjective measurements and promises to make long-term studies more common.
(2) Is it possible to tame the vastness of the fatigue field and the many medical conditions associated with it, by searching for common root causes such as aging? Current studies could benefit from broadly interdisciplinary collaborations. For example, stratifying healthy subjects based on their epigenetic markers of aging, as opposed to their calendar ages, may provide new insights for interpreting behavioral metrics.
(3) Is “mind over matter” a real thing? Work presented in this Research Topic and our previous work (Urquhart et al., 2019, 2020), among other published studies, would suggest that the answer is, likely, yes. The younger, physically fitter, and possibly more motivated you are, the more likely you are to recruit frontal networks that drive conscious effort to compensate for fatigue in sensorimotor areas.

In all, it feels like this field is in its infancy. It will be exciting to see what the future brings.

## Author Contributions

GA prepared this article. HN, DM, and CP have reviewed and have approved it for publication. All authors contributed to the article and approved the submitted version.

## Conflict of Interest

The authors declare that the research was conducted in the absence of any commercial or financial relationships that could be construed as a potential conflict of interest.

## Publisher's Note

All claims expressed in this article are solely those of the authors and do not necessarily represent those of their affiliated organizations, or those of the publisher, the editors and the reviewers. Any product that may be evaluated in this article, or claim that may be made by its manufacturer, is not guaranteed or endorsed by the publisher.
